# The Optimization of the Debittering Process and the Exploration of Bitter Metabolites of *Paeonia ostii* ‘Fengdan’ Seeds

**DOI:** 10.3390/plants14020198

**Published:** 2025-01-12

**Authors:** Shuting Li, Yanfeng Xu, Xinyue Liu, Qizhen Su, Junyu Zhang, Xinran Zhang, Xinmiao Guo, Yanlong Zhang, Qingyu Zhang

**Affiliations:** 1 College of Landscape Architecture and Arts, Northwest Agriculture and Forestry University, Yangling 712100, China; lishuting@nwafu.edu.cn (S.L.); barronxu@nwafu.edu.cn (Y.X.);; 2 Qinling National Botanical Garden, Xi’an 710061, China; 3 Longchi Peony Industry Co., Ltd., Heze 274000, China

**Keywords:** tree peony seed, bitterness, LC-ESI-QQQ-MS, polyphenolic metabolites

## Abstract

Tree peony seeds, traditionally used for edible oil production, are rich in α-linolenic acid (ALA). However, little attention is paid to their development as a healthcare food due to their bitter and astringent taste. The aim of this study was to optimize the debittering process of peony seeds on the basis of maintaining nutritional value and to identify the compounds that cause the taste of bitterness. We first optimized the debittering process by orthogonal experiments which reduced the polyphenol content by 90.25%, and we measured the main nutritional value of fatty acid composition, indicating that the high content of ALA is not affected by debittering. Then, we identified and determined the types and content of polyphenols, the metabolites causing bitter taste, in the samples based on LC-ESI-QQQ-MS. Principal component analysis (PCA) and orthogonal partial least square discriminant analysis (OPLS-DA) were used to compare and analyze the seeds at different stages of debittering. Thirty-eight key metabolites were identified, of which paeoniflorin, taxifolin, alibiflorin, protocatechuic acid, benzoyl paeoniflorin, quercetin-*3*-galactoside, and oxpaeoniflorin were significantly compared, and most of them were positively correlated with bitter taste. These results are conducive to the exploration and study of the bitter taste and nutritional value of tree peony seeds in the future.

## 1. Introduction

Tree peony (*Paeonia* section Moutan DC.), a traditional Chinese flower, has gained increased attention in recent years due to its new role as a woody oil crop [1,2,3]. Tree peony seeds are rich in unsaturated fatty acids (USFAs) that are essential for human health, including more than 40% omega-3 USFAs [4]. In addition, tree peony seeds also contain high levels of liposoluble substances, such as *β*-sitosterol, apigenin, squalene shark, *β*-carotene, and plant polyphenols [5], which endow tree peony seeds with multiple health benefits, including antioxidant, lipid-lowering, liver protection, immune regulation, blood glucose control, anti-inflammatory, and cytotoxic activities [6,7,8]. Although tree peony seed oil (TPSO) is available on the market, the low oil yield and complex extraction process keep the price of TPSO relatively high, and the target consumer group is relatively narrow [9]. If tree peony seeds, which are plump and large, can be made into nut-type healthcare products for people, the utilization efficiency of the seeds will be greatly improved, and the low-end consumer market will be opened up.

Fengdan peony (*Paeonia ostii* T Hong et J × Zhang, hereinafter referred to as ‘Fengdan’) is a widely cultivated variety of oil peony in China [10]. In addition, the content of USFAs, especially *α*-linolenic acid (ALA), in the seeds of ‘Fengdan’ is among the best in tree peony, making it imperative to develop ‘Fengdan’ as an important woody oil crop [11]. However, in the process of turning ‘Fengdan’ seeds into a new healthcare food, bitterness will become a major limiting factor affecting their flavor, which will reduce the satisfaction in taste and restrict the choice of food manufacturers and consumers. Therefore, understanding the metabolite composition causing bitterness is of great help in converting ‘Fengdan’ seeds into novel dietary supplements.

Bitterness is prevalent in many plants, and polyphenols are the most abundant substances that contribute to bitterness in plants [5]. To utilize the nutritional value of plants, people often strive to minimize bitterness as much as possible, and thus have actively explored debittering processes for plants [12,13,14,15]. However, there is limited research on the debittering process of tree peony seeds. In view of the great differences in the debittering treatment in different plants and the influence factors considered, there is no systematic process that can be used for the debittering of tree peony seeds. Utilizing the characteristic that polyphenols can be dissolved in acidic liquids or alcohols, plant tissues and organs can be soaked in acidic or alcoholic solutions for debittering [16], providing a new approach for optimizing the bitterness removal process of tree peony seeds.

High-Performance Liquid Chromatography (HPLC) or Ultra-High-Performance Liquid Chromatography (UHPLC) is the primary method for detecting polyphenols in plants. Andrewes et al. [17] analyzed polyphenol compounds in virgin olive oil by using reversed-phase HPLC and performing sensory evaluation, and they identified 12 main peaks of polyphenol compounds. The sensory evaluation indicated that all detected polyphenol compounds contributed to the bitter taste. Curko et al. [18] investigated two grape varieties and analyzed the composition of proanthocyanidins and anthocyanins, which belong to polyphenols, using HPLC-UV-FluO/MS. The results showed a positive correlation between the content of proanthocyanidins and bitterness in grape seeds. To investigate the bitter polyphenol compounds in chocolates and cocoa beans, Fayeulle et al. [19] used HPLC-QQQ-MS as the detection method, and the results revealed that flavan-3-ols, which are epicatechin derivatives, were the main substances causing the bitter sensation. In addition to polyphenols, many other metabolites, such as alkaloids, amino acids, polypeptides and saponins, have also been detected to induce a bitter flavor in various plants [20,21,22,23,24]. Regarding tree peony, although it has been reported that there are abundant secondary metabolites in seeds [25], the compounds responsible for bitterness have not received sufficient attention.

We hope to develop a debittering process of ‘Fengdan’ seeds, so that it can have the unique nutritional value of high ALA content in the form of a nut-type food after flavor improvement in the future, and we hope to explore the types of polyphenols that cause bitter taste through the determination of polyphenols before and after debittering. In this study, we first conducted a three-factor and three-level orthogonal experiment to explore the debittering process of ‘Fengdan’ kernels. Then, we compared the nutritional quality of ‘Fengdan’ kernels before and after the debittering, especially the ALA content, to explore whether the debittering process would affect the ALA content. Further, we determined the changes in the polyphenol content during the debittering process by Liquid Chromatography–Electrospray Ionization–Triple Quadrupole Mass Spectrometry (LC-ESI-QQQ-MS); this was performed to identify the polyphenols that cause the bitter taste of ‘Fengdan’ seeds, thereby providing a theoretical basis for the study of debittering technology, the nutritional value of tree peony seeds, and the development of healthcare food.

## 2. Results and Discussion

### 2.1. Acidic Solution Soaking Reduces the Total Polyphenol Content of Paeonia ostii ‘Fengdan’ Kernels

Orthogonal experiment has been widely used in the optimization of conditions for the determination of plant secondary metabolites [26,27,28,29]. In this study, the kernels were soaked in acidic solution to debitter, and pH, temperature, and processing time were selected as the experimental factors.

**Figure 1:**
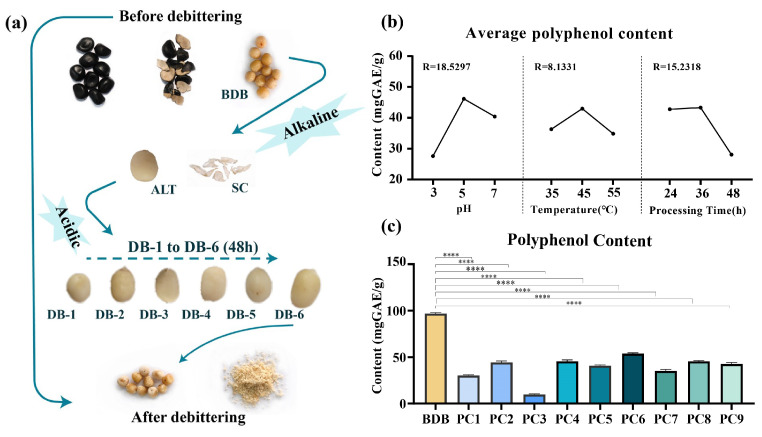
Result and analysis of orthogonal experiment. (**a**) The process of the bittering, (**b**) range analysis of the orthogonal experiment on the effect of factors in seeds debittering, and (**c**) polyphenol content of each processing combination in the orthogonal experiment. BDB: seed samples of before debittering; ALT: seed samples of alkaline solution treatment; SC: seed endopleura; DB-1: debittered 8 h samples; DB-2: debittered 16 h samples; DB-3: debittered 24 h samples; DB-4: debittered 32 h samples; DB-5: debittered 40 h samples; DB-6: debittered 48 h samples. **** indicates the significance at the level of 0.01%.

According to the results of the orthogonal experimental design table, nine experimental treatment combinations were conducted to probe into the effects of different pH values (3, 5, 7), temperatures (35 °C, 45 °C, 55 °C), and processing times (24 h, 36 h, 48 h) on the debittering effect. The polyphenol content, which was measured by the Folin phenol reagent method, represented the bitterness, and the reduction in bitter taste was determined by sensory evaluation. The conditions of each combination and the polyphenol content of DB-6 kernels were presented in Table 1. In order to illustrate the debittering effect of each group, the polyphenol content of the nine combinations was compared with that of the BDB ‘Fengdan’ kernels (Figure 1a). The results indicated that the polyphenol content of each treatment group was significantly reduced, and the effect of combination 3 (PC3) was the optimum, with the polyphenol content being 90.25% lower than that of the BDB kernels (Figure 1c). This result was confirmed by the sensory evaluation of kernels before and after suffering, that is, the bitterness of PC3 was the weakest (Table S4). Correlation analysis also showed that the scores of sensory analysis for each PC were significantly positively correlated with the polyphenol content (Table S5 and Figure S3).

### 2.2. Effect of Each Factor/Level on Debittering

Range analysis was employed to determine the effect of different levels in each factor on the polyphenol content (Figure 1b and Table 1). In range analysis, the range (R) value was calculated to reflect the volatility of each factor on the debittering results. According to the R value calculation results, the degree of influence of each factor on the effect of debittering was in the order of pH (18.5297), processing time (15.2318), and temperature (8.1331); pH and processing time in particular had the best effect on the reduction in polyphenol content (Table 1). There were differences on the debittering effect in the same factor. In terms of pH, the content of polyphenols at level 1 (pH = 3) was the lowest, indicating that pH = 3 has the best debittering effect. As for temperature and processing time, the debittering effect was better at level 3 (55 °C and 48 h). Among the three factors, level 2 had the worst effect on bitterness removal.

The correlation among each factor and the results were further verified through variance analysis (Table 2). According to the analysis results, the high F-values of pH and processing time indicated that these two factors were more significantly associated with the reduction in polyphenol content than the temperature. The *p* value also revealed the same result. From the perspective of statistical significance, pH and treatment time had significant effects on the polyphenol content (*p* < 0.05), while the result of treatment temperature (*p* = 0.08 > 0.05) suggested that temperature was not an important factor affecting the polyphenol content of seeds. Therefore, acidity and treatment time have a substantial influence on debittering in the process optimization of ‘Fengdan’ kernels.

Taken together, the best process for eliminating bitterness from ‘Fengdan’ kernels is to soak the kernels in acidic solution with pH = 3 and treat them at 55 °C for 48 h. The optimal process conditions obtained by range analysis are precisely in line with combination 3 of the orthogonal test.

Bitterness impacts the taste and commodity value of foods; thus, the debittering process is a research topic of interest for many researchers who focus on the edibleness of plants. Kore and Chakraborty [15] investigated the debittering process of pummelo juice by adjusting syrup concentration, pH, and hot water pretreatment, and the results indicated that the addition of syrup mitigated bitter taste. However, in the debittering process of lupin, treatment with 0.5% NaHCO_3_ had a better effect than the high-temperature water soaking treatment [30]. Fontoin et al. [14] probed the effects of pH, tartaric acid concentration, and ethanol level on the astringency and bitterness of red wine through three single-factor experiments. In summary, pH, solution–material ratio, temperature, processing time, addition of sweetener, etc., can become the research conditions of the debittering process, and different choices will be made considering the diverse characteristics of plants. Although we are not certain about the contribution to debittering of other conditions in this study, there are a few studies on the optimization of the debittering process of ‘Fengdan’ kernels through orthogonal experiments, which can offer an effective experimental reference for exploring the comprehensive influence of various conditions on the experimental results.

In the existing research on the debittering process, we found that the use of chemical agents is not the only way to remove bitterness [13,29]. In order to better compare the effect of different processes of debittering on ’Fengdan’ kernels, ultrasonic debittering and heating debittering were applied. The total polyphenol content of ’Fengdan’ seeds decreased by 79.56% compared with that before the removal of bitterness, after the peony seeds were mixed with water at a ratio of 1:5 and ultrasounded (45/80 KHz) for 60 min. After ultrasonic treatment for 60 min, the total polyphenol content of ’Fengdan’ kernels was decreased by 79.56% compared with that of BDB kernels (Figure S2a). Meanwhile, when the kernels were treated at 70 °C for 6 h, the content of total polyphenols decreased by 60.18% (Figure S2b). Although these two methods can also significantly reduce the content of total polyphenols, the debittering effect is not the best compared with the optimized acidic solution treatment in the present study. However, the processing time of ultrasonic and heating is short, and the health concerns caused by chemical reagent residues can be avoided, so further exploration of these two debittering processes in actual production is expected to provide a better choice for debittering.

### 2.3. Effect of Debittering on Morphological Characteristics of Paeonia ostii ‘Fengdan’ Kernels

The seeds of *Paeonia ostii* ‘Fengdan’ are composed of two layers of seed coat (testa and endopleura) and the kernel. The ‘Fengdan’ kernels typically present a fawn color, and the kernels after debittering appear relatively light in color compared to that before debittering (Figure 1a). The surface of the kernels after debittering occasionally has a layer of white substance sticking to it, which is due to the residue of pectin. The longitudinal diameter (LD) of the seed is measured along the direction of the germ, and the transverse diameter (TD) is measured along the direction perpendicular to the LD, and these two were measured to signify the size of the seed. Additionally, the weight of the seed is indicated by the hundred-grain weight of the seeds.

**Figure 2:**
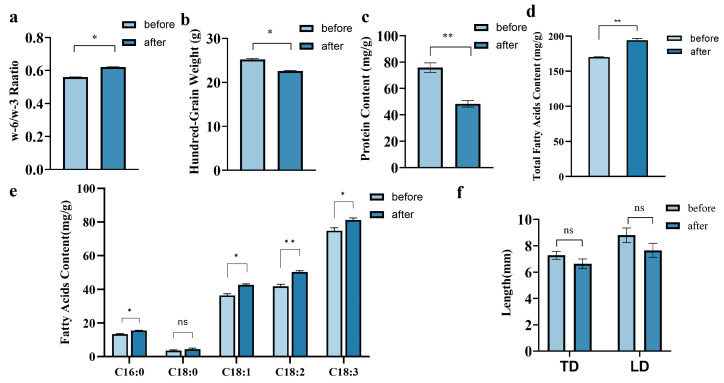
Morphological characteristics and nutritional qualities of BDB and DB-6 ‘Fengdan’ kernels. (**a**) Fatty acid radio, (**b**) hundred-grain weight of BDB and DB-6 kernels, (**c**) protein content of kernels, (**d**) total fatty acid content, (**e**) fatty acid composition of BDB and DB-6 ‘Fengdan’ kernels, (**f**) comparison of transverse and longitudinal diameters of kernels. * indicates the significance at 5% level, ** indicates the significance at 1% level.

The results demonstrated that there was no significant difference between the TD and LD of ‘Fengdan’ kernels before and after debittering, suggesting that the debittering process had no effect on the size (Figure 2f). However, the hundred-grain weight of DB-6 ‘Fengdan’ kernels decreased by 10.35% compared with that of BDB (25.11 g), indicating that the process caused some material loss of ’Fengdan’ seed (Figure 2b).

### 2.4. Effect of Debittering on Some Nutritional Qualities of Paeonia ostii ‘Fengdan’ Kernels

High ALA content provides ‘Fengdan’ seeds with a distinctive human-health application value. The results showed that the content of total fatty acids in DB-6 kernels has significantly increased, from 170.21 mg/g to 194.21 mg/g (Figure 2d). Regarding the content of main five fatty acids, as shown in Figure 2e, the content of *α*-linolenic acid increased from 74.88 mg/g to 81.23 mg/g, and other important fatty acids, including palmitic acid (C16:0), stearic acid (C18:0), oleic acid (C18:1), and linoleic acid (C18:2), were increased to varying degrees. The ratio of 18:2/18:3, that is, ω-6/ω-3, increased slightly, changing from 0.5598 to 0.6201, indicating that the relative content of ω-6 fatty acids increased, while that of ω-3 decreased (Figure 2a).

In terms of protein content, it decreased by 42.29%, from 79.21 mg/g to 45.71 mg/g (Figure 2b,c). Combined with the reduction in hundred-grain weight, it was inferred that the debittering process caused the loss of some nutrients including protein in ‘Fengdan’ kernels, but had little effect on the composition of fatty acids.

The decrease in the relative content of ALA (ω-3 fatty acids) might be related to its chemical characteristics. Previous studies have shown that USFA, including α-linolenic acid, are prone to oxidation under high temperature, resulting in the loss of USFA [31]. The temperature of soaking treatment involved in the debittering process is not high compared to that of the oxidation condition, so it is speculated that the temperature treatment in debittering may have a negative but not significant impact on the content of ALA, and the overall ALA remains at a high level. The high content of ALA provides a low ω-6/ω-3 ratio, which is of great significance in optimizing human fatty acid intake to improve health, to TPSO. Although the debittering process slightly improves this ratio, it still has an advantage in fatty acid composition compared to common vegetable oils [32]. However, it is worth noting that the comparison of protein content before and after debittering in this study shows that the protein content of ‘Fengdan’ kernels decreases significantly, while in previous studies, no significant change in protein content was found in the debittering process as in this study [30,33], which may be due to the influence of acidic solution soaking on protein storage.

Although the ω-6/ω-3 ratio showed a decrease in ALA content relative to LA, the overall fatty acid content, including ALA, was significantly increased in the seeds after debittering (Figure 2d,e). Combined with the decrease in seed weight and protein content after debittering, this indicated that acid soaking may not lead to the loss of fatty acids but other nutrients, including protein, to reduce seed weight, resulting in an increase in the content of fatty acids in seeds. In addition, the total fatty acid content of the seeds measured prior to bitterness removal in this study was 170.21 mg/g, which is lower than the 191 mg/g reported in a previous study, but remains largely consistent. ALA content also decreased correspondingly [11]. Specifically, the ALA content measured in the seeds before debittering was 74.88 mg/g, accounting for approximately 44% of the total fatty acids, aligning closely with the ALA composition in *P. ostii*. These findings suggest that the fatty acid profiles of the seeds analyzed in this study are consistent with those of *P. ostii* seeds under standard conditions. However, it should be noted that the fatty acid content in plants may vary due to environmental and climatic factors across different years.

To sum up, after debittering, the unique nutritional value of peony seed with high ALA content can still be maintained.

### 2.5. Analysis of Bitter Metabolites in Paeonia ostii ‘Fengdan’ Seeds

#### 2.5.1. Bitter Metabolite Statistical Analysis

**Figure 3:**
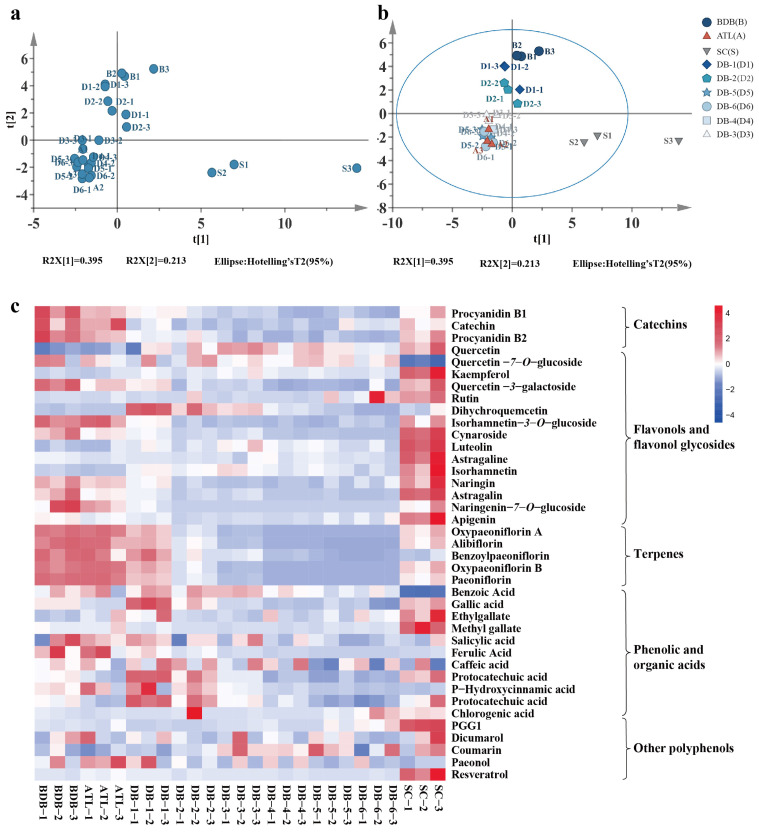
Multivariate statistical analysis of pods and seeds during development. (**a**) The PCA score scatter plot, (**b**) the OPLS-DA score scatter plot. (**c**) Heat map of metabolite level in kernel debittering process (BDB, ALT, DB-1, DB-2, DB-3, DB-4, DB-5, DB-6) and endopleura (SC). ALT: endopleura removed stage. DB-1 to DB-6: acid solution treatment stage. The red and blue boxes indicate values above and below the average, respectively.

The polyphenols in the BDB and DB-6 ‘Fengdan’ kernels were separated with the LC-ESI-QQQ-MS system (Figure S4). To make a visual comparison of seeds focused on quantitative data of targeted compounds, a principal component analysis (PCA) model was generated (Figure 3a). The PCA score scatter plot showed significantly separated trends between seeds. Then, an orthogonal partial least square–discriminant analysis (OPLS-DA) model was established, which enhanced the separated trend by decreasing unrelated noise in the data (Figure 3b).

**Figure 4:**
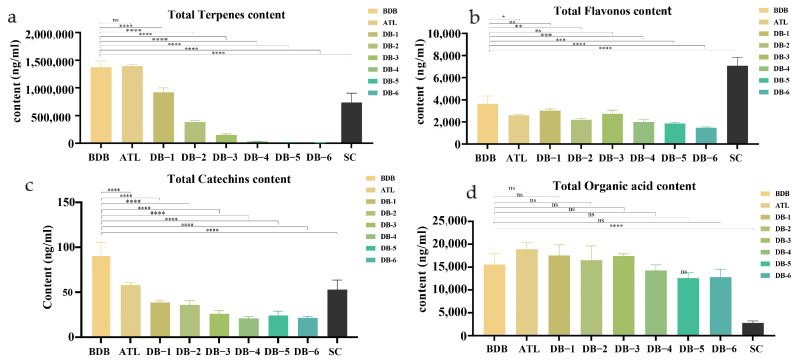
Changes in metabolite content in the debittering process of ‘Fengdan’ kernels. (**a**) Total terpene content, (**b**) total flavonoid content, (**c**) total catechin content, (**d**) total organic acid content (*p* < 0.05). * indicates the significance at 5% level, ** indicates the significance at 1% level, *** indicates the significance at 0.1% level, **** indicates the significance at 0.01% level.

A heat map based on the detected polyphenols provided an overview of the differences in polyphenol contents between the BDB and DB-6 processes’ kernels, and endopleura (SC) was also involved (Figure 3c). A total of 38 phenolic compounds were identified, mainly divided into four categories (Figure 4), including 18 flavonoids, 5 phenolic derivatives of mon oterpenes, 10 organic and phenolic acids, 2 biscoumarins, resveratrol, prostaglandin G1 (PGG1), and other polyphenols (Figure 3 and Table S1). Changes in the contents of these detected polyphenols during the debittering process were analyzed.

#### 2.5.2. Differentially Accumulated Metabolites in Debittering

Through the above analysis, it was found that there were differences in phenolic metabolites in the seeds during acid solution treatment, according to VIP > 1, log2(foldchange) > 1, and *p* < 0.05 from the *t*-test (Figure S5). Analyzing the metabolites of different comparison groups by volcano plot, we detected 18 (2 upregulated and 16 downregulated), 18 (2 and 16), 16 (4 and 12), 16 (4 and 12), 10 (3 and 7), and 3 (2 and 1) phenolic metabolites that exhibited differential abundance in ALT vs. DB-6, DB-1 vs. DB-6, DB-2 vs. DB-6, DB-3 vs. DB-6, DB-4 vs. DB-6, and DB-5 vs. DB-6 comparison groups, respectively (Figure S5a–f). Analysis showed that metabolite changes were mainly concentrated in the periods of ALT and DB-3. We found seven kinds of phenolic metabolite differences between samples, namely, paeoniflorin, taxifolin, alibiflorin, protocatechuic acid, benzoyl paeoniflorin, quercetin-*3*-galactoside, and oxpaeoniflorin.

### 2.6. Specific Phenolic Metabolite Analysis

#### 2.6.1. Monoterpene Glycosides

The main monoterpene glycosides detected in ‘Fengdan’ seeds were paeoniflorins, including paeoniflorin, albiflorin, benzoylpaeoniflorin, oxypaeoniflorin A, and oxypaeoniflorin B. Paeoniflorin, which is volatile, always decomposes when exposed to heat, acids, and alkalis, so the change in paeoniflorin during the debittering treatment is significant. The content of paeoniflorin and total paeoniflorins before debittering accounted for a large proportion in ‘Fengdan’ kernels, and the change was the most obvious (Figure 4a).

Paeoniflorin was the metabolite with the highest content of polyphenols in ‘Fengdan’ seeds detected in this study. It was the first extracted from the root of tree peony and is a characteristic component of *Paeoniaceae* plants [34]. Paeoniflorin has high pharmacological value and plays a role in immune regulation, the regulation of Ca^2+^ and ROS homeostasis, and alleviating neurodegeneration, has anti-inflammatory and anti-depression activity, and so on [35,36,37]. Therefore, ‘Fengdan’ seeds, which are rich in paeoniflorin, have unique research value in food applications. In this study, although the content of paeoniflorin was significantly reduced by the debittering process, it was still higher than other polyphenols. Therefore, we deduced that paeoniflorin was one of the metabolites causing bitterness, but the residual amount after debittering could still maintain the healthcare value of ‘Fengdan’ seeds.

#### 2.6.2. Flavonoids

**Figure 5:**
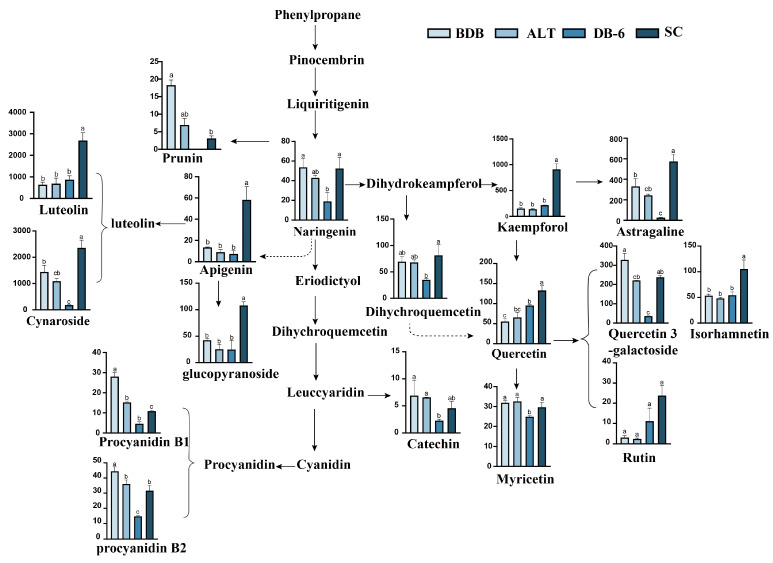
Differences in the flavonoid pathway metabolites among BDB, ALT, DB-6, and SC. The *y*-axis represents the MS intensity based on LC-MS. a, b, c lowercase letters in each graph indicate significant differences among cultivars (*p* < 0.05).

Flavonoid compounds are the most diverse and varied metabolites in the process of debittering ‘Fengdan’ kernels (Figure 5).

##### Quercetins

In ‘Fengdan’ seeds, quercetin, as well as its conjugated forms with glycosides or methyl, such as isorhamnetin and rutin, was detected. The quercetin content increased first and then decreased, reaching a peak at 36 h during the debittering treatment, and then gradually declined. However, the quercetin content still increased from 46 ng/mL to 95.2 ng/mL at the end of debittering, and some quercetin and its derivatives also existed in the endopleura.

As one of the important kinds of flavonoids, quercetins are widely distributed in fruits and vegetables such as onions and mangoes [38]. Previous studies have concluded that quercetins are important metabolites affecting bitterness in chrysanthemum [39] and tartary buckwheat [40]. However, in this study, the content of quercetins was not reduced by the debittering process, so it was deduced that quercetins are not the key compounds causing bitterness in ‘Fengdan’ seeds.

Quercetins and its derivates exhibit abundant biological activities. Quercetin has anticancer activity as an inhibitor of human cathepsin B [41], and quercetin works against Ebola virus [42]. There are also other methylated derivatives with different activities. For example, rhamnetin (7-Methoxylated quercetin) has anti-inflammatory actions [43]. Given the result that the content of quercetin and part of its derivatives were not affected by debittering, the healthcare function of ‘Fengdan’ seeds was effectively maintained.

##### Luteolin and Kaempferol

Luteolin exists mostly in the form of glucosides (luteolin-7-glucoside) in plants [44]; LC-MS results showed that luteolin-7-glucoside decreased significantly during the debittering process.

Luteolin, which has strong antioxidant, anti-inflammatory, and diabetic biological effects [45,46,47,48], is commonly used as an antioxidant in biochemistry and is found in chrysanthemum, spinach, carrots, citrus, and other plants. We detected high luteolin content in ‘Fengdan’ kernels and found that luteolin decreased during debittering, so we speculated that luteolin may affect the flavor of ‘Fengdan’ seeds as a bitter compound.

##### Catechins

Catechins, which are widely found in green tea and betel nut, are precursors of tannins [48]. Three kinds of catechins were detected, namely catechin, procyanidin B1, and procyanidin B2. The content of procyanidin B2 was relatively high but decreased from 51.35 ng/mL to 14.63 ng/mL after debittering. In addition, the contents of catechin and procyanidin B1 were also lower than those before bitterness removal, and some of them were distributed in the endopleura.

Procyanidins (PAs) are natural flavan-3-olpolymers that protect plants from biological and abiotic stresses and are beneficial to human health, but in flavor, like catechins, they impart bitterness and astringency to foods [49,50]. We detected the presence of catechins in ‘Fengdan’ seeds, and their content decreased due to debittering. Catechins have strong antioxidant activity function. The phenolic hydroxyl groups in the structure of catechins can react with free radicals. They themselves will be oxidized to form relatively stable phenoxy radicals, thus terminating the free radical chain reaction and protecting cells from free radical damage. Combined with previous studies, catechins should be important metabolites that affect the bitterness of tree peony seeds.

#### 2.6.3. Phenolic and Organic Acids

In this study, 10 organic acids were identified, with the total content decreased. Among them, the relative content of benzoic acid was the highest, and there was no significant change in the content of benzoic acid before and after debittering, and most of them existed in the seed kernels. Benzoic acid, a balm from *Liquidambar orientalis*, is commonly used as a flavoring and fragrance fixing agent, and it is speculated that it may alleviate the bitter taste in tree peony seeds [51].

The contents of ferulic acid, p-hydroxycinnamic acid, and protocatechuic acid were lower than before debittering treatment. Ferulic acid has been proven to have various biological activities and is a kind of phenolic acid beneficial to human health, and its taste is mostly bitter [52].

#### 2.6.4. Resveratrol

Most of the resveratrol existed in the inner seed coat, and a small amount existed in the kernel, and its content did not change significantly. Resveratrol is a polyphenol stilbene compound, which is an antibiotic, and its anticancer, antibacterial, antioxidant, and other healthcare functions have been verified in wine [53,54].

## 3. Materials and Methods

### 3.1. Materials

#### 3.1.1. Seed Materials

Seeds of *Paeonia ostii* ‘Fengdan’ were used as research materials in this study. The seeds were collected from 8-year-old ‘Fengdan’ (4–6% moisture content in seed) preserved in the Peony Germplasm Resource Nursery of Northwest A&F University (34°15′ N, 108°03′ E, and Alt. 448 m) in Yangling, China. After manually removing the hard testa of seeds, the mature kernels were used for the study of the debittering process and the compositions of bitter metabolites.

#### 3.1.2. Chemicals and Reagents

AR-grade HCl and NaOH (for debittering process) and Folin reagent (for polyphenol content determination) were purchased from GuangHua Sci-tech (Shantou, China). HPLC-grade methanol, sulfuric acid, toluene, and n-hexane for fatty acid component analysis and LC-ESI-QQQ-MS analysis were purchased from XiLong Science (Shantou, China).

### 3.2. Debittering Process of Paeonia ostii ‘Fengdan’ Seeds

The method of acid and alkali soaking was used to remove the bitterness of ‘Fengdan’ seeds.

#### 3.2.1. Removal of Inner Seed Coat

A ‘Fengdan’ kernel is covered by a thin inner seed coat, and the kernel and the endopleura are closely adhered to each other under the action of pectin. The presence of an inner seed coat can affect the extraction of polyphenols, so the endopleura needs to be removed before debittering. The pectin will decompose when it is met with alkali. The mature kernels were placed in alkaline solution (adjusted with 1 mM NaOH, pH = 9) and then bathed in water at 100 °C for 15 min. After the water bath, the kernels were rinsed with water and rubbed gently to peel the inner seed coat.

#### 3.2.2. Debittering by Acidic Solution

The best debittering treatment for ‘Fengdan’ kernels was studied by the orthogonal experiment of three factors and levels. The orthogonal experimental design is shown in Table 3.

Solutions with different acidity levels (adjusted with 1 mM HCl, pH = 3, pH = 5, and pH = 7) were prepared with acetic acid (food grade), and the seeds of ‘Fengdan’ were mixed with the acid solution at 1:5 (*w*/*v*), and then treated with a constant-temperature water bath at different temperature conditions (35 °C, 45 °C, and 55 °C) for different amounts of time (24 h, 36 h and 48 h), and the acidic solution was replaced every 12 h during debittering. Since we hope to develop the kernel into a nut-type food, we retained the whole kernel without any grinding treatment during the debittering process. After debittering, the ‘Fengdan’ seeds were rinsed with water and then dried at 50 °C to prevent mildew.

#### 3.2.3. Determination of Total Polyphenol Content

The total polyphenol content of ‘Fengdan’ kernels was determined by the Folin phenol regent method [55]. In the establishment of the standard curve, the linear fitting equation of absorbance (y) and gallic acid concentration (x) of the sample was obtained as y = 0.0029x + 0.0865 (R2 = 0.9984).

Then, 3 g of ‘Fengdan’ kernels was weighed and grinded in a grinder (JXFSTPRP-24, SHANGHAIJINGXIN, Shanghai, China) for 30 s, and then extracted in an ultrasound bath (45/80 KHz; KQ-500DE, KunshanShuMei, Kunshan, China) at 70 °C for 40 min (1:5 *w*/*v* seeds: water). Then, 1 mL was absorbed from the extraction solution, 5.0 mL of water was added, 1.0 mL of sodium tungstate–sodium molybdate mixed solution and 3.0 mL of Na_2_CO_3_ solution were added, and they were left for 2 h for color development. The absorbance was determined at the wavelength of 765 nm and taken into the standard curve to calculate the final polyphenol content of the extraction solution, then converted into the final total polyphenols of ‘Fengdan’ kernels (mgGAE/g).

#### 3.2.4. Sensory Evaluation of Bitter Taste of ‘Fengdan’ Kernel

To ensure that changes in polyphenol content affected the intensity of bitterness, volunteers were recruited to perform sensory evaluations of kernels from different treatment groups in the orthogonal experiment.

A total of 20 volunteers participated in this study, comprising 6 teachers and 14 students majoring in this field. The age range for the teachers was 32 to 40 years, while that for the students was 20 to 28 years. Both groups maintained a balanced gender ratio of 1:1. All participants were in good health and had not experienced any illness within the week preceding the sensory evaluation. Additionally, they did not consume any medication, excitatory drinks (such as alcohol, tea, and coffee), or spicy foods two hours prior to the test. And they avoided engaging in any vigorous physical activity two hours before the test.

Each volunteer first randomly selected 3 kernels without debittering for tasting and then randomly selected 3 seeds from each treatment group for tasting. Before tasting each kernel, the mouths of the volunteers were rinsed to make sure they were clean and free of other flavors. At the end of the tasting, a score was given on a scale of 1 to 10, with a lower score indicating less bitterness and a higher score indicating a taste more similar to the bitterness before debittering.

### 3.3. Nutritional Quality Evaluation Before and After Debittering

#### 3.3.1. Determination of Fatty Acids’ Composition

Quantitative fatty acid analysis was carried out by GC-MS (8890A/5975C, Agilent Technologies, Shanghai, China); the GC was equipped with a G4513A automatic sampler and a 19091N-1361 GC column. The operating condition referred to the method published by Zhang [11,56].

In order to ensure the stability of the FAs, the preparation of fatty acid methyl esters (FAMEs) was carried out. Sulfuric acid–methanol was used as a methylating reagent. Briefly, 4 mL of methanol containing 5% concentrated sulfuric acid and 1.2 mL of toluene were used to resuspend dried lipids by 2 h of incubation in a 85 °C water bath (Thermomix, Eppendorf, Hamburg, Germany). After cooling to room temperature, 2 mL of deionized water was added to terminate the reaction. FAMEs were subsequently extracted with 2 mL of n-hexane and centrifuged (1000 rpm for 10 min), and the upper phase was then collected and 1 μL was injected for GC-MS analysis.

Ultra-high-purity helium was used as the carrier gas (with the flow rate of 1.0 mL min^−1^) and FA composition analysis was performed in constant flow mode. The injector temperature was set at 250 °C for split injection at a split ratio of 10:1. And the temperature of the quadrupole, ion source, and transfer line were set at 150, 230, and 280 °C, respectively. The total run time was 40 min: the oven temperature rose to 120 °C for 1 min, then increased to 175 °C at 10 °C min^−1^ and was held for 10 min, then rose to 210 °C at 5 °C min^−1^ for 5 min, and then rose to 230 °C for 5 min. The FA qualitative analysis was achieved by comparing the mass spectra to those available in the database (NIST08 Library) and co-elution with corresponding standards. A standard curve method with an internal standard was used as a quantitative approach to construct five calibration plots of analyte/internal standard peak area ratio vs. standard concentration, as determined by the least squares method. FAMEs in each sample were measured using methyl heptadecanoate as the internal standard and expressed as milligrams per gram DW of a sample. All samples were analyzed in triplicate.

#### 3.3.2. Quantification of Soluble Protein Content

Coomassie brilliant blue was used to determine the soluble protein content of each sample, repeated 3 times, and the average value was taken. A standard curve, made by the concentration gradient of bovine serum protein, was used to calculate the protein content. Then, 0.5 g of leaf sample was weighed, SiO_2_ and distilled water were added into the homogenate, and it was cleaned with distilled water and then collected in a 10 mL centrifuge tube. Centrifuging took place at 8000 r/min for 10 min, and supernatant was collected for determination. Then, 1 mL of the extraction solution was taken and 5 mL of Coomassie brilliant blue G250 solution [0.01% (*w*/*v*) Coomassie brilliant blue G250, 4.7% ethanol (*v*/*v*), 8.5% phosphoric acid (*w*/*v*)] was added and quickly shaken well; it was left for 2 min and then the absorbance was determined at a 595 nm wavelength. The content of soluble protein was measured by a standard curve (y = 0.003x + 0.468, R^2^ = 0.994).

### 3.4. Identification of Polyphenol Compounds in Paeonia ostii ‘Fengdan’ Seeds During Debittering

#### 3.4.1. Preparation of Seed Samples

The mature seeds of *Paeonia ostii* ‘Fengdan’ were collected from the tree Peony Research Center of Northwest A&F University and hulled mechanically. Samples were taken every 8 h during the 48 h debittering process. Nine groups were set, namely, before debittering (BDB), alkaline solution treatment (ALT), during the process of debittering (DB-1, DB-2, DB-3, DB-4, DB-5, DB-6), and endopleura (SC), with three replicates in each group.

The seed samples were stored at −80 °C until analysis. Before the analysis, seeds of tree peony were dried using the LGJ-10D vacuum freeze dryer (Sihuan Scientific Instrument Factory, Beijing, China). Approximately 0.3 g of the samples was extracted using 2 mL 80% (*v*/*v*) of methanol with the aid of 40 kHz ultrasonic treatment at 30 °C for 30 min. The mixture was centrifuged with 13,363× *g* at 4 °C for 5 min using a KDC-140HR centrifuge (ZhongkeZonkia Scientific Instruments, Hefei, China). After repeated extraction and centrifugation 3 times, the supernatant was mixed. The mixture was filtered through a 0.22 μm filter and transferred into LC bottles for LC analysis.

#### 3.4.2. LC-ESI-QQQ-MS Analysis of Methanol Extract

The chromatographic separation was performed using an ExionLC™ AC (AB SCIEX, Framingham, MA, USA) system with an InertSustain AQ-C18 (4.6 mm × 150 mm, 5 μm, Shimadzu, Japan) column. Eluent A was an aqueous H_2_O solution of 0.1% HCOOH, and eluent B was methanol. The elution gradient was implemented as follows: 25% B at 0 min, 25% B at 1 min, 95% B at 7 min, 95% B at10 min, and 25% B at 10.5 min. The flow rate was set to 0.7 mL min^−1^. The column temperature was maintained at 40 °C. The injection volume was 2 μL for each sample.

The MS analysis was performed in a triple quadrupole 5500 mass spectrometer (AB SCIEX, Framingham, MA, USA). The ion source temperature (T) was set to 600 °C, the ion spray voltage (IS) was set to 4.5 kV, the curtain gas was set to 35 mL min^−1^, the nebulizer gas (GS1) was set to 60 mL min^−1^, and the heater gas (GS_2_) was at 65 mL min^−1^. Nitrogen was used as GS_1_, GS_2_, and curtain gas. Multiple reaction monitoring (MRM) was used for mass separation as well as analyte detection. Data were analyzed using Analyst 1.7 and MultiQuant 3.0.2 software (AB SCIEX, Framingham, MA, USA) [57]. Concentrations of the corresponding metabolites were determined according to a calibration curve established from the corresponding chemical standards (Tables S2 and S3).

### 3.5. Statistical Analysis

Range analysis and variance analysis were used to analyze the results of orthogonal experiments [58]. K values and R values must be calculated in range analysis. The K value is the sum of all experimental values at the same level within the same factor, and then the average K value, that is, the average experimental value at the same level within the same factor, needs to be calculated. The R value is the value difference between the maximum K-mean value and the minimum. The contribution of different factors to results is analyzed by comparing the size of R values.

The SPSS 25.0 was used for the statistical data analysis of variance analysis of orthogonal experiments, phenotypic characteristic and nutritional quality of ‘Fengdan’ kernels. In variance analysis, Duncan’s multiple-range test was used to separate means. Significance was accepted at *p* < 0.05 throughout the analysis, and analyzed results were the average value of triplicate measurements on the duplicate samples. Statistical analyses of the FA composition and protein content were tested by one-way ANOVA analysis (*p* < 0.05) and comparisons between means were performed with Tukey′s test. All experiments were performed with three replicates.

Quantitative dates of the seed debittering stage were imported into SIMCA14.1 for multivariate statistical analysis, and the principal component analysis model was generated and the orthogonal partial least squares discriminant analysis was performed to analyze the relationship between the samples.

## 4. Conclusions

Tree peony seeds, which possess high *α*-linolenic acid content, have the potential to be developed into health products. However, their bitterness will impact the taste; therefore, it is requisite to optimize the process of seed debittering and recognize the bitter metabolites of tree peony seeds. In this study, the kernels of *Paeonia ostii* ‘Fengdan’ were taken as the research object, and the optimized debittering process of ‘Fengdan’ was obtained. Soaking the kernels in an acid solution with pH = 3 and treating them at 55 °C for 48 h significantly reduced the total polyphenols of the seeds, and the bitter taste of the ‘Fengdan’ seeds could be effectively eliminated. The LC-ESI-QQQ-MS system was utilized to detect polyphenols, which are the main compounds causing the bitter taste, in the ‘Fengdan’ seeds before and after debittering. The results indicated that paeoniflorin, taxifolin, alibiflorin, protocatechuic acid, benzoyl paeoniflorin, quercetin-*3*-galactoside, and oxpaeoniflorin were significantly reduced after debittering, suggesting that these substances all contributed to the bitter taste. Paeoniflorin, a special secondary metabolite in tree peony, also decreased significantly, indicating that paeoniflorin might be a unique compound involved in the bitterness of tree peony. Additionally, the nutritional characteristics of the ‘Fengdan’ kernels before and after debittering demonstrated that the process did not have a significant impact on the important nutrient, *α*-linolenic acid, of tree peony seeds and the high nutritional value of unsaturated fatty acids was well maintained.

## Figures and Tables

**Table 1:** Results of orthogonal experiment.

Processing Combinations	pH	Temperature (°C)	Processing Time (h)	Polyphenol Content (mgGAE/g)
1	3	35	24	29.9253 ± 1.1078
2	3	45	36	43.5259 ± 2.0622
3	3	55	48	9.3822 ± 1.0635
4	5	35	36	44.6006 ± 2.1256
5	5	45	48	40.4397 ± 1.1195
6	5	55	24	53.3822 ± 1.1474
7	7	35	48	34.4310 ± 2.0603
8	7	45	24	45.0201 ± 1.2101
9	7	55	36	41.8218 ± 2.098
K1	27.6111	36.3190	42.7759	
K2	46.1408	42.9952	43.3161	
K3	40.4243	34.8621	28.0843	
R	18.5297	8.1331	15.2318	

**Table 2:** Results of variance analysis of all factors in this orthogonal experiment.

Factors	Degrees of Freedom (df)	Sum of Squares (s)	Variance	F Value	*p* Value	Significance
pH	2	1979.092	989.546	27.474	0.000	**
Temperature (°C)	2	207.096	103.548	2.875	0.080	ns
Processing Time (h)	2	1153.083	576.542	16.007	0.000	**

** indicates significant at 1% level.

**Table 3:** The factors and levels of the debittering conditions.

Levels	Factors		
	A-pH	B-Temperature/°C	C-Processing Time/h
1	3	35	24
2	5	45	36
3	7	55	48

## Data Availability

The original contributions presented in the study are included in the article; further inquiries can be directed to the corresponding author.

## Supplementary Materials

The following supporting information can be downloaded at https://www.mdpi.com/article/10.3390/plants14020198/s1: Table S1: Total identified metabolites in tree peony fruit; Table S2: Validation data for the quantitative HPLC-MS/MS method; Table S3: MS parameters of samples. Table S4: Sensory evaluation of debittering by orthogonal experiment; Table S5: Correlation analysis between total polyphenol content in orthogonal experiment and bitterness score in sensory evaluation; Figure S1: *Paeonia ostii* ‘Fengdan’ seeds’ phenotype chart; Figure S2: Polyphenol content of ultrasonic debittering and heating debittering; Figure S3: A linear fit between total polyphenol content in orthogonal experiment and bitterness score in sensory evaluation; Figure S4: Fingerprint of polyphenols; Figure S5: Differentially accumulated metabolites in debittering.
