# Supplementary material for: The Optimization of the Debittering Process and the Exploration of Bitter Metabolites of Paeonia ostii ‘Fengdan’ Seeds
Source: Plants (Basel). 2025 Jan 12;14(2):198. doi: 10.3390/plants14020198 (PMC11769413; doi:10.3390/plants14020198)

Table S1. Total identified metabolites in tree peony fruit.

ID	RT (min)	Assigned identity	Molecular formula	[M-H]-	Fragment ions	Calculated Conc.(ng/ml)									class	Identification
						BDB	ALT	DB-1	DB-2	DB-3	DB-4	DB-5	DB-6	SC		
**1**	3.38	Procyanidin B1	$C_{30}H_{26}O_{12}$	577	407	24.49±6.4	15.28±0.66	10.91±0.64	9.16±2.15	6.78±0.56	5.59±1.79	5.95±1.59	4.66±0.93	12.98±3.58	Flavonoids	GNPS
**2**	4.27	Gallic acid	$C_7H_6O_5$	169	125	100.29±0	62.12±3.14	208.22±33.86	106.05±21.42	61.76±2.49	49.08±1.91	54.22±9.92	30.35±21.11	129.37±16.86	Phenolic acids	[1]
**3**	3.64	(+)-Catechin	$C_{15}H_{14}O_7$	289	203	8.92±3.99	8.76±3.8	2.63±1.11	0.82±0.7	0.8±0.5	1.26±0.41	1.67±2.25	2.28±0.27	4.57±1.25	Flavonoids	GNPS;[2]
**4**	4.67	Ethylgallate	C_9H_{10}O3	197	124	37.08±0.01	37.21±0.1	37.3±0.17	37.12±0.02	37.11±0.03	37.17±0.11	37.13±0.04	37.22±0.06	37.52±0.3	Phenolic acids	GNPS;[2]
**5**	3.36	Methyl gallate	$C_8H_8O_5$	183	124	11.44±4.4	16.24±10.81	7.86±3.5	4.62±0.4	4.08±0.44	4.53±0.73	4.7±1.67	6.39±1.78	83.12±31.98	Phenolic acids	[2]
**6**	3.39	procyanidin B2	$C_{30}H_{26}O_{12}$	576.9	407	51.35±12.18	36.08±2.59	24.68±1.16	25.88±4.4	18.33±3.69	15.39±2.18	16.36±3.09	14.64±0.41	35.3±6.9	Flavonoids	GNPS
**7**	3.35	Oxypaeoniflorin A	$C_{23}H_{28}O_{12}$	495.2	137	24044.13±1293.34	23558.49±1199.94	16168.81±1664.21	5819.19±1430.24	1637.04±264.23	411.7±47.35	207.65±29.72	140.16±50.6	14285.14±3462.15	Terpenes	[1]
**8**	4.08	Albiflorin	$C_{23}H_{28}O_{11}$	479	449.2	983971.8±110189.91	896265.89±179004.83	665131.43±64672.67	327776.31±75084.83	113820.1±13461.4	24891.87±2437.39	11593.56±1435.52	8490.24±3148.54	542151.29±120622.54	Terpenes	[2]
**9**	5.53	benzoylpaeoniflorin	$C_{30}H_{32}O_{12}$	583	121.2	321.07±38.6	276.48±102.32	285.03±52.37	123.58±43.94	109.73±13.45	53.26±6.84	32.09±11.37	20.12±3.41	72.95±27.57	Terpenes	GNPS
**10**	3.34	Hydroxypaeoniflorin	$C_{23}H_{26}O_{10}$	495.2	136.9	26565.7±1783.21	27871.09±735.08	19605.42±2313.82	7907±2141.02	2283.41±354.39	552.12±70.22	283.17±32.84	193.72±74.54	17045.13±4169.79	Terpenes	GNPS;[1]
**11**	4.11	Paeoniflorin	$C_{23}H_{28}O_{11}$	479	121.2	339996.63±15758.97	341485.23±29040.04	217974.69±12998.05	92010.87±15474.87	31875.48±5551.59	7458.08±1059.76	3778.18±712.9	2683.55±933.01	163572.15±39572.67	Terpenes	GNPS;[2]
**12**	5.62	Quercetin 7-O-glucoside	$C_{21}H_{20}O_{12}$	433.1	271.2	46.06±16.94	65.51±13.07	86.5±51.09	107.35±27.25	148.83±5.84	120.23±17.74	120.94±12.75	95.21±2.37	152.97±35.82	Flavonoids	[2]
**13**	4.31	Quercetin	C15H10O7	301	179	10.54±0.41	10.42±0.15	10.31±0.36	10.5±0.25	10.36±0.09	10.37±0.13	10.47±0.08	10.37±0.15	9.52±0.18	Flavonoids	[1]
**14**	5.58	Kaempferol	$C_{15}H_{10}O_6$	285	142.9	169.86±33.49	174.23±62.4	282.61±46.07	229.81±32.6	353.15±84.5	262.77±32.88	257.54±53.22	236.73±35.61	1166.29±451.45	Flavonoids	TCM
**15**	4.26	Quercetin 3-galactoside	$C_{21}H_{20}O_{12}$	477	301	357.87±57.32	200.56±36.03	187.68±15.29	94.4±38.91	101.08±11.54	47.41±10.68	54.58±10.98	73.21±64.8	295.34±100.64	Flavonoids	TCM
**16**	3.99	Rutin	$C_{27}H_{30}O_{16}$	609	271	3.12±1.06	3.68±2.12	2.56±0.72	6.41±7.43	2.13±0.72	1.92±1.04	7.06±7.83	27.16±28.11	23.85±4.94	Flavonoids	GNPS;[1]
**17**	4.85	Taxifolin	$C_{15}H_{12}O_7$	303	285	69.31±9.22	68±4.63	436.41±35.24	278.24±90.28	217.81±19.96	115.64±21.59	75.4±16.97	34.82±2.3	114.45±58.84	Flavonoids	GNPS
**18**	5.01	Myricetin	$C_{15}H_{10}O_8$	317	179	31.9±1.06	32.56±2.06	27.5±0.36	24.86±0.61	24.75±0.27	24.53±0.42	24.66±0.75	24.89±0.52	29.65±2.29	Flavonoids	GNPS
**19**	4.24	Cynaroside	$C_{21}H_{20}O_{11}$	449.1	153.1	1689.17±467.37	999.33±168.25	702.6±87.93	405.34±99.42	457.48±149.61	195.67±38.98	213.2±63.62	177.63±17.88	2778.06±762.21	Flavonoids	TCM
**20**	5.59	Luteolin	$C_{15}H_{10}O_6$	285.2	133	640.06±123.06	693.15±256.34	1066.55±162.3	876.45±134.58	1334.06±278.83	1061.25±121.03	984.15±174.07	770.76±179.65	2984.21±567.49	Flavonoids	TCM; GNPS
**21**	4.51	Apigenin	$C_{15}H_{10}O_5$	269	151.1	13.55±0.33	9.06±2.35	9.64±0.32	9.16±1.24	8.43±8.15	12.86±6.74	10.65±3.01	7.43±3.05	81.29±35.45	Flavonoids	TCM
**22**	4.03	Isorhamnetin	$C_{16}H_{12}O_7$	315.2	150.9	53.52±3.07	48.33±1.61	75.49±6.37	71.44±4.6	81.89±8.22	64.96±5.37	67.14±4.4	54.37±6.29	147.16±72.98	Flavonoids	[1]
**23**	4.31	Naringin	$C_{27}H_{32}O_{14}$	579	271	53.64±9.35	42.95±2.69	30.18±4.09	12.86±4.55	7.87±1.3	8.36±1.86	10.33±5.96	18.99±8.96	79.4±47.41	Flavonoids	TCM; GNPS
**24**	4.23	Astragaline	$C_{21}H_{20}O_{11}$	447	284.1	415.94±156.5	225.15±34.61	148.91±16.43	81.96±30.28	94.29±34.62	39.93±1.64	44.77±16.94	34.09±14.06	681.94±194.36	Flavonoids	GNPS
**25**	4.5	Naringenin-7-O-glucoside	$C_{21}H_{22}O_{10}$	431.1	268	13.19±8.88	6.95±1.83	1.88±1.05	0.68±0.06	0.65±0.47	1.56±0.45	1.35±0.23	1.38±O.10	5.18±3.64	Flavonoids	GNPS
**26**	4.51	Isorhamnetin-3-O-glucoside	$C_{22}H_{22}O_{12}$	315.2	315.2	42.28±3.06	25.22±9.06	22.09±6.07	6.31±2.78	7.72±3.67	6.63±2.07	11.14±11.75	24.66±17.63	166.94±102.17	Flavonoids	GNPS
**27**	5.65	Benzoic Acid	$C_7H_6O_2$	120.9	77	14853.72±2354.35	14892.81±5258.12	16621.59±2353.18	15894.75±3086.31	16913.22±474.33	13880.31±1247.23	12235.1±1203.77	12504.12±1696.58	2133.75±378.65	Phenolic acids	Standard
**28**	5.85	salicylic acid	$C_7H_6O_3$	137.3	92.8	172.72±64	165.68±11.41	169.44±9.81	135.33±35.89	154.85±32.25	135.89±18.35	129.2±12.79	124.02±10.06	137.16±21.75	Phenolic acids	[1]
**29**	4.99	Ferulic Acid	$C_{10}H_{10}O_4$	193	134	94.41±76.34	94.14±69.03	42.14±13.42	24.29±7.32	15.41±2.35	8.21±2.78	7.83±0.26	6.59±0.99	11.85±2.51	Phenolic acids	TCM; GNPS;
**30**	4.01	Caffeic acid	$C_9H_8O_4$	179	134.9	1.59±0.14	1.44±0.42	1.75±0.47	1.73±0.38	1.55±0.53	1.67±0.5	1.07±0.44	1.39±0.68	1.22±0.79	Phenolic acids	TCM;[1]
**31**	3.79	protocatechuic acid	$C_7H_6O_4$	153	109	67.43±6.75	39.23±11.87	139.65±6.1	92.99±25.71	54.84±0.42	32.35±8.95	25.08±11.62	24.48±3.85	99.92±20.62	Phenolic acids	TCM
**32**	4.84	p-Hydroxycinnamic acid	$C_9H_8O_3$	463	300	107.53±17.05	136.75±110.54	218.51±228.04	147.94±39.78	69.93±8.23	37.35±10.58	19.24±2.31	11.73±2.34	14.76±6.98	Phenolic acids	TCM
**33**	3.55	Chlorogenic acid	$C_{16}H_{18}O_9$	353	191	15.54±1.88	13.22±1.78	13.41±0.12	126.63±16.18	14.26±1.41	13.33±0.19	15.66±2.59	32.8±10.74	26.95±1.69	Phenolic acids	TCM
**34**	4.13	PGG1	$C_{20}H_{34}O_6$	939	769.1	259.71±41.73	271.7±181.59	103.33±27.49	118.84±31.11	221.84±63.88	210.24±153.17	363.13±246.51	673.76±213.46	2609.69±294.29	Fatty acids	TCM
**35**	9.88	Dicumarol	$C_{19}H_{12}O_6$	335	161	10.68±0.08	10.69±0.11	10.69±0.04	10.64±0.03	10.69±0.11	10.65±0.03	10.69±0.03	10.61±0.01	10.76±0.14	Phenylpropanoids	TCM; GNPS
**36**	6.45	Coumarin	$C_9H_6O_2$	147	77	3.51±1.95	2.33±1.64	2.9±0.87	4.16±0.52	8.1±2.48	6.32±0.53	8.44±2.96	4.8±4.99	6.81±3.12	Coumarins	TCM
**37**	8.47	Paeonol	$C_9H_{10}O_3$	167	121.1	39.54±26.63	78.4±28.87	52.56±45.23	25.28±11.56	21.6±22.52	35.4±21.02	12.79±5.71	24.41±22.27	13.37±6.02	Phenols	TCM;[1]
**38**	5.4	Resveratrol	$C_{14}H_{12}O_3$	227	143	645.29±132.59	1238.43±324.36	894.6±32.6	568.03±277.3	736.38±71.92	606.38±97.05	446.01±11.7	451.92±77.96	6850.67±1677.2	Stilbenes	GNPS; TCM;

RT = retention time; GNPS = Global Natural Products Social Molecular Networking platform; TCM = Traditional Chinese Medicine library. All the targeted compounds were confirmed by chemical standards.

Wang X, Li C, Contreras MDM, Verardo V, Gómez-Caravaca AM, Xing C. Integrated Profiling of Fatty Acids, Sterols and Phenolic Compounds in Tree and Herbaceous Peony Seed Oils: Marker Screening for New Resources of Vegetable Oil.

Foods. 2020 Jun 11;9(6):770.

Li, S.S.; Wu, Q.; Yin, D.D.; Feng, C.Y.; Liu, Z.A.; Wang, L.S. Phytochemical variation among the traditional Chinese medicine Mu Dan Pi from Paeonia suffruticosa (tree peony). Phytochemistry 2018, 146, 16-24

Table S2. Validation data for the quantitative HPLC-MS/MS method.

Standards	Calibration equation	R^2	Standards	Calibration equation	R^2
Procyanidin B1	y=1503.26527x+1739.54976	0.99968	Hyperoside	y=7702.53248x+1.34079e5	0.99933
Gallic acid	y=9192.08311x+-9.43532e4	0.99934	Luteolin	y=19957.45995x-1407.02205	0.99998
(+)-Catechin	y=356.65458x+4127.10329	0.99907	Apigenin	y=18455.61591x-3.45863e4	0.99996
Ethylgallate	y=1182.19279x+10417.13061	0.99975	Isorhamnetin	y=18919.28105x-1.70425e5	0.99918
Methyl gallate	y=8.26990e4x-4.45445e5	0.99981	Astragaline	y=10228.68558x+4.75030e4	0.99968
Procyanidin B2	y=285.95267x+494.06193	0.99953	Naringenin-7-O-glucoside	y=3.59793e4x+1.54625e5	0.99946
Oxypaeoniflorin A	y=4860.54397x+20393.04163	0.99911	Isorhamnetin-3-O-glucoside	y=3219.05162x-4875.25878	0.99968
Albiflorin	y=491.54189x+8491.20584	0.99924	Benzoic acid	y=1747.45523x+3.71276e4	0.99964
Benzoyl paeoniflorin	y=873.15055x-2095.59914	0.99929	Salicylic acid	y=4.03186e4x+5.46009e5	0.99993
Hydroxypaeoniflorin	y=4860.54397x+20393.04163	0.99911	Ferulic acid	y=3065.47044x+8722.47982	0.9998
Paeoniflorin	y=287.00181x+7276.21591	0.99993	Caffeic acid	y=6.37849e4x-22115.60222	0.99992
Quercetin 7-O-glucoside	y=5.91180e4x+1.62030e5	0.99875	Protocatechuic acid	y=19600.47019x-7.88038e4	0.99955
Quercetin	y=28379.84990x-3.72299e5	0.99912	p-Hydroxycinnamic acid	y=11791.14139x+3.45215e4	0.99936
Kaempferol	y=1022.67822x+841.36289	0.99996	Chlorogenic acid	y=14186.13453x-1.60415e5	0.99312
Quercetin 3-galactoside	y=10772.41991x-1.05603e5	0.99273	PGG1	y=40.24267x-1937.41498	0.987782
Rutin	y=4255.42414x-125.23387	0.99759	Dicumarol	y=1556.71955x-952.11773	0.99715
Taxifolin	y=11198.57532x-18545.40135	0.99987	Coumarin	y=9700.52295x+12087.08261	0.99583
Myricetin	y=5239.98953x-1.24538e5	0.9995	Paeonol	y=3075.84245x+7265.47743	0.9992
Cynaroside	y=4.32803e4x-1.64262e5	0.9981	resveratrol	y=3065.47044x+8722.47982	0.9998

Table S3. MS Parameters of samples.

Assigned identity	CXP	EP	DP	CE	Assigned identity	CXP	EP	DP	CE
Procyanidin B1	11.00	10.00	-187.00	-31.00	Luteolin	11.00	10.00	-18.16	-34.43
Gallic acid	11.00	10.00	-27.41	-19.38	Apigenin	11.00	10.00	-28.13	-33.04
(+)-Catechin	11.00	10.00	-35.99	-29.99	Isorhamnetin	11.00	10.00	-27.00	-19.00
Ethylgallate	11.00	10.00	-70.00	-30.00	Naringin	11.00	10.00	-18.01	-32.92
Methyl gallate	11.00	10.00	-80.07	-27.37	Astragaline	11.00	10.00	-21.94	-37.91
procyanidin B2	11.00	10.00	-43.19	-29.95	Naringenin-7-O-glucoside	11.00	10.00	-32.11	-42.68
Oxypaeoniflorin A	11.00	10.00	-73.91	-29.90	Isorhamnetin-3-O-glucoside	11.00	10.00	-250.00	-38.06
Albiflorin	11.00	10.00	-56.96	-20.48	Benzoic Acid	11.00	10.00	-52.02	-16.06
benzoylpaeoniflorin	11.00	10.00	-70.40	-22.72	salicylic acid	11.00	10.00	-99.31	-25.85
Hydroxypaeoniflorin	11.00	10.00	-73.91	-29.90	Ferulic Acid	11.00	10.00	-67.52	-17.39
Paeoniflorin	11.00	10.00	-61.91	-18.30	Caffeic acid	11.00	10.00	-73.13	-20.85
Quercetin 7-O-glucoside	11.00	10.00	-32.11	-32.08	protocatechuic acid	11.00	10.00	-27.41	-19.38
Quercetin-3-O-glucuronide	11.00	10.00	-90.00	-39.00	p-Hydroxycinnamic acid	11.00	10.00	-90.00	-39.00
Kaempferol	11.00	10.00	-25.86	-50.03	Chlorogenic acid	11.00	10.00	-64.06	-21.86
Quercetin 3-galactoside	11.00	10.00	-123.01	-28.14	PGG1	11.00	10.00	-10.00	-45.00
Rutin	11.00	10.00	-91.17	-47.81	Dicumarol	11.00	10.00	-47.67	-17.04
Taxifolin	11.00	10.00	-20.04	-17.10	Coumarin	11.00	10.00	-33.38	-17.29

Myricetin	11.00	10.00	-94.83	-26.97	Paeonol	11.00	10.00	85.00	25.00
Cynaroside	11.00	10.00	-18.89	-18.08	Resveratrol	11.00	10.00	-31.22	-26.03

Table S4. Sensory evaluation of debittering by orthogonal experiment

**PC No.**	PC1	PC2	PC3	PC4	PC5	PC6	PC7	PC8	PC9
Volunteer 1	4	6	3	5	6	8	6	6	6
Volunteer 2	4	7	2	6	7	7	8	6	6
Volunteer 3	5	7	2	8	4	6	7	7	6
Volunteer 4	4	4	1	6	8	8	5	7	6
Volunteer 5	3	5	1	5	5	7	6	8	8
Volunteer 6	6	8	2	5	5	9	6	6	7
Volunteer 7	4	4	3	8	6	7	7	7	8
Volunteer 8	6	6	4	7	6	7	7	6	7
Volunteer 9	5	5	2	5	4	5	8	6	7
Volunteer 10	5	4	3	5	5	8	8	6	7
Volunteer 11	3	6	2	4	7	8	8	6	6
Volunteer 12	4	5	3	6	6	7	8	5	7
Volunteer 13	4	6	2	6	3	8	6	8	8
Volunteer 14	6	5	3	7	7	8	5	9	5
Volunteer 15	5	4	3	6	5	7	6	7	6
Volunteer 16	5	5	4	5	8	7	7	6	5
Volunteer 17	4	5	2	4	5	5	6	5	5
Volunteer 18	6	8	1	5	5	8	6	8	6
Volunteer 19	4	4	1	7	7	9	8	6	5
Volunteer 20	3	7	1	8	5	8	8	6	5
**Average score***	4.5	5.55	2.25	5.9	5.7	7.35	6.8	6.55	6.3

* The total score of sensory evaluation is 10 points. A low score indicates a weak bitterness, and a higher score indicates a closer resemblance to the bitterness before debittering.

Table S5. Correlation analysis between total polyphenol content in orthogonal experiment and bitterness score in sensory evaluation

	**Pearson correlation index**	***P***
**Value**	0.904	<0.001

Supplementary Figure S1 *Paeonia ostii* ‘Fengdan’ seeds Phenotype Chart. (a) *Paeonia ostii* ‘Fengdan’ seeds phenotype, (b) Phenotypic changes of *Paeonia ostii* ‘Fengdan’ seeds before and after debittering.

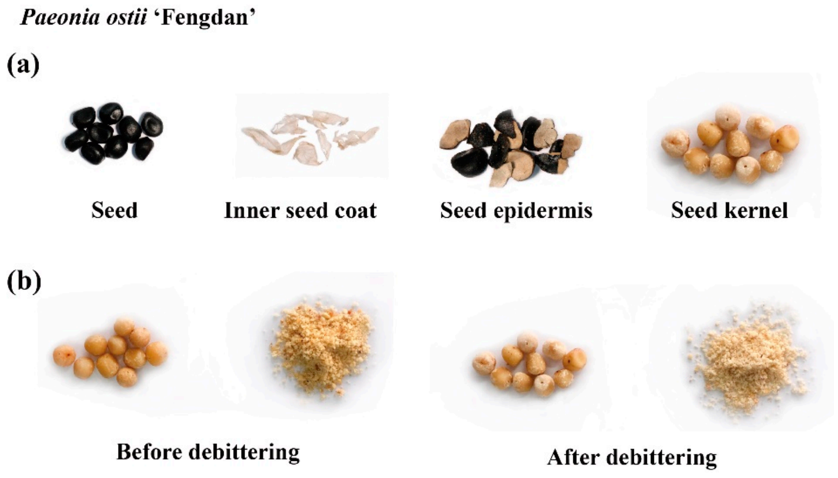

Supplementary Figure S2. Polyphenol content of ultrasonic debittering and heating debittering. (a) Results of polyphenol content showed the best effect of ultrasonic debittering for 60min. U-30/45/60 indicates ultrasonic treatment for 30/45/60 minutes. (b) The content of polyphenol in ‘Fengdan’ kernels was significantly reduced by heating debittering.

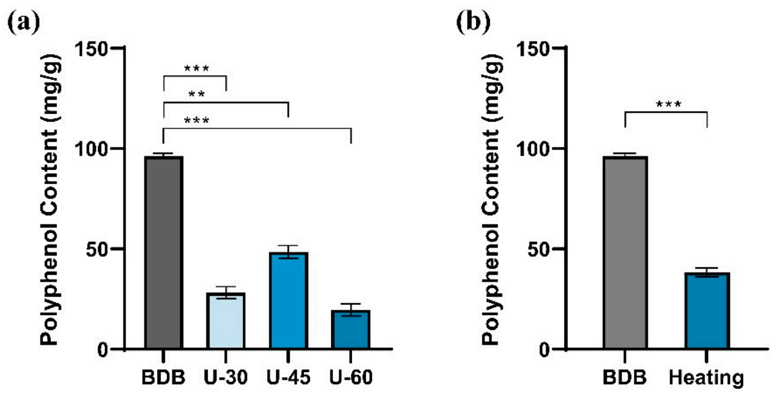

Supplementary Figure S3. A linear fit between total polyphenol content in orthogonal experiment and bitterness score in sensory evaluation

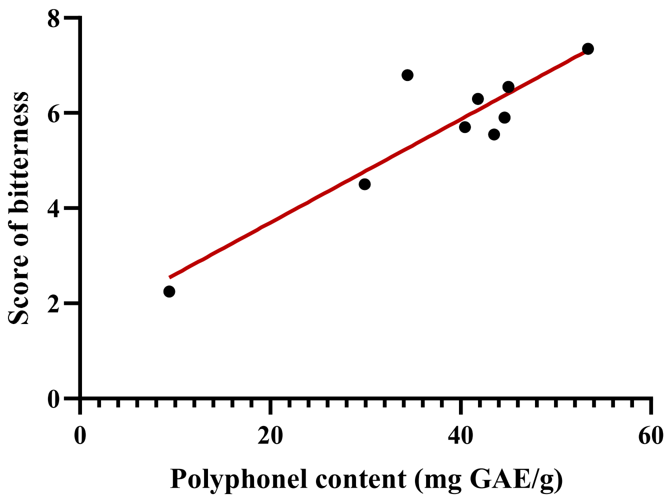

Supplementary Figure S4. Fingerprint of polyphenols

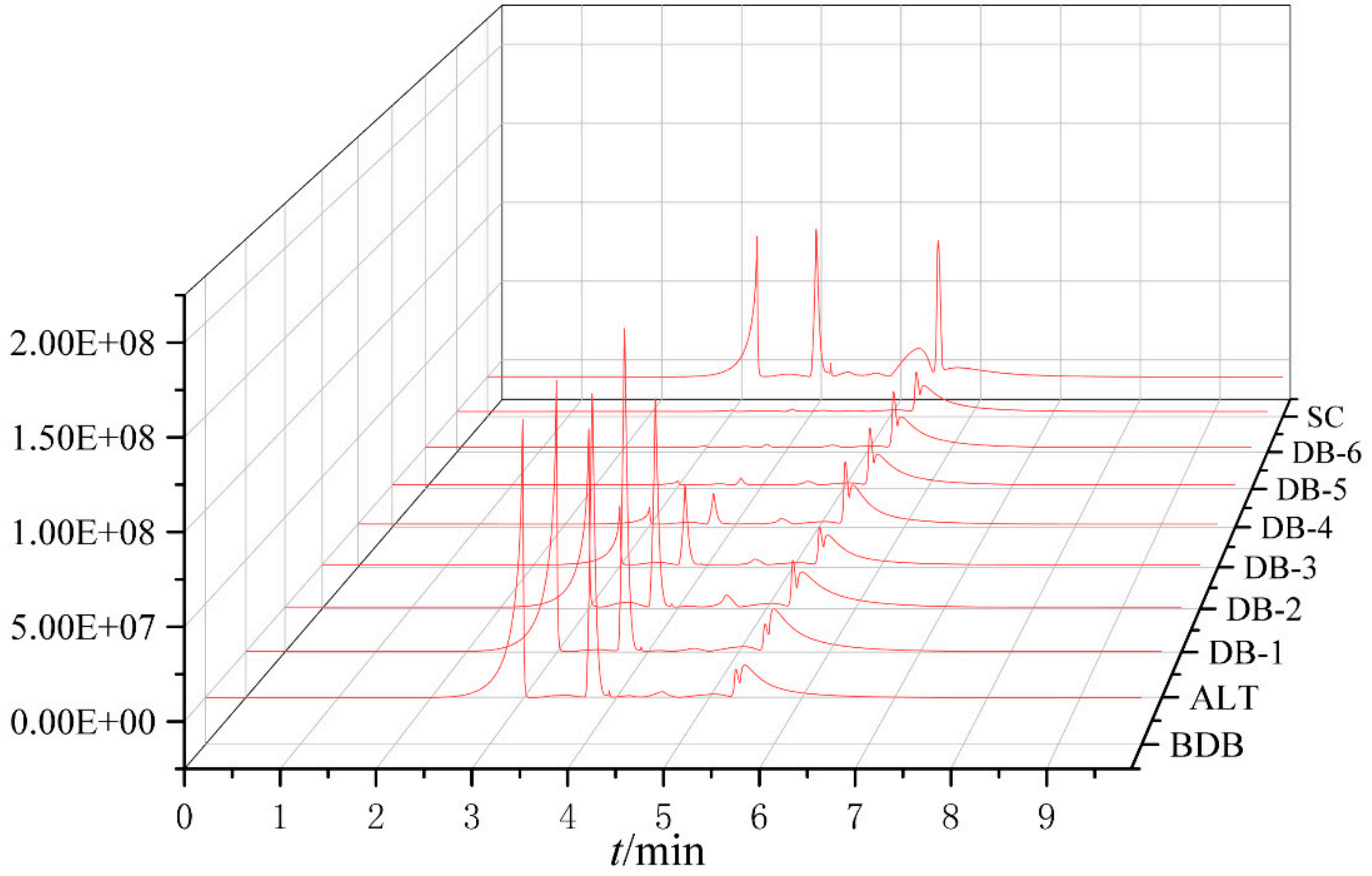

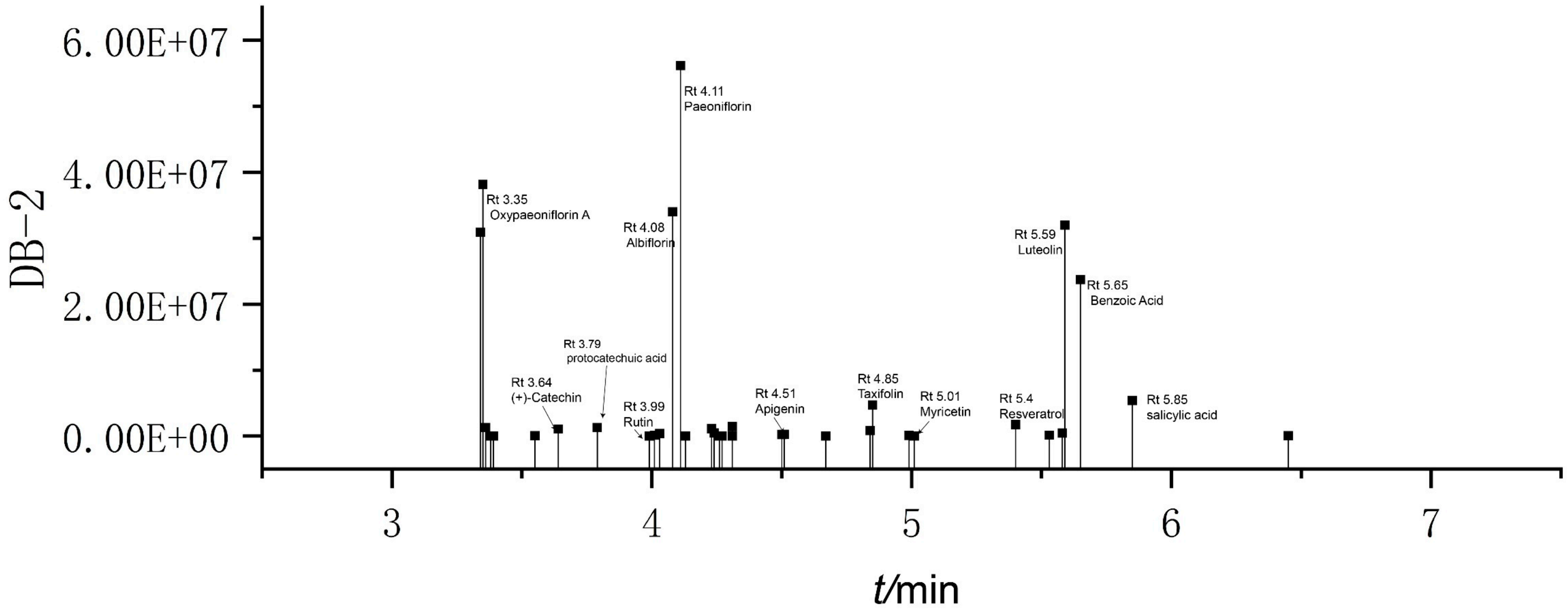

Supplementary Figure S5. Differentially Accumulated metabolites in the debittering. (a-f) volcano plot of different comparison groups (p < 0.05).

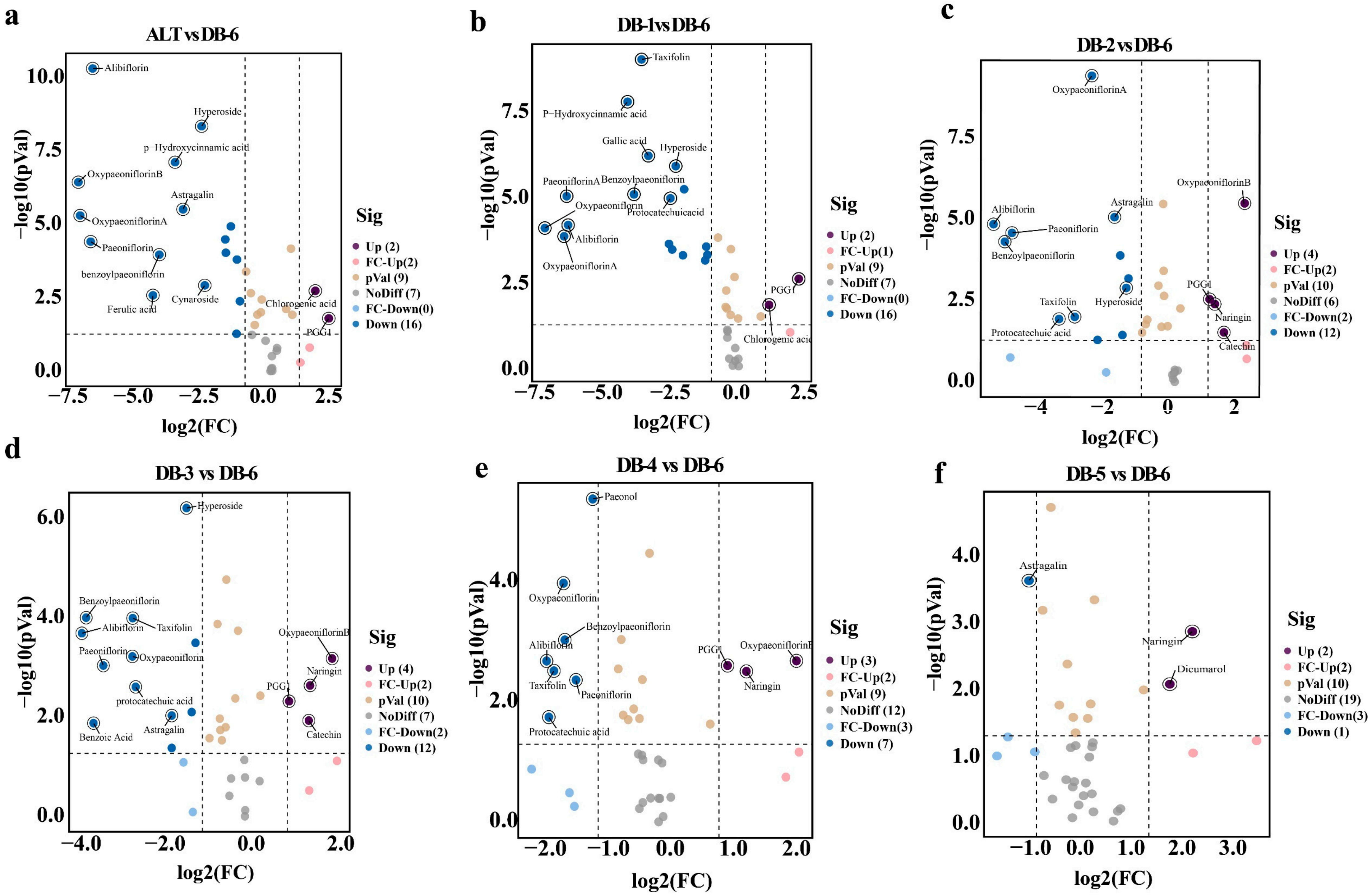

Supplement: Supplementary file 1 [file plants-14-00198-s001.zip › plants-3319136-supplementary.pdf]
